# A Reversion Spectroscope

**Published:** 1871-03

**Authors:** 


### A REVERSION SPECTROSCOPE.

An important addition to the resources of spectrum analysis has been made by Zollner's invention of a reversion spectroscope, by which extremely small changes of refrangibility, and consequently comparatively slow motions of a star or sun-flame, can be detected. It consists of a spectroscope in which, by reflection, the spectrum of a source of light can be superposed above a reversed spectrum of the same source; so that if a white flame containing sodium be viewed, there will be seen in the upper part of the field a sodium line with the blue end of the spectrum on the one side, and underneath it a sodium line with the red end of the spectrum on the same side. The two bright lines may be made to coincide exactly by an adjustment; and, if any change in refrangibility takes place, the motion of the line is doubled, and is also more exactly measured, because it is referred to itself as a standard.—*English Mechanic.*

